# Natural History of Malignant Bone Disease in Gastric Cancer: Final Results of a Multicenter Bone Metastasis Survey

**DOI:** 10.1371/journal.pone.0074402

**Published:** 2013-10-28

**Authors:** Nicola Silvestris, Francesco Pantano, Toni Ibrahim, Teresa Gamucci, Fernando De Vita, Teresa Di Palma, Paolo Pedrazzoli, Sandro Barni, Antonio Bernardo, Antonio Febbraro, Maria Antonietta Satolli, Paola Bertocchi, Vincenzo Catalano, Elisa Giommoni, Alessandro Comandone, Evaristo Maiello, Ferdinando Riccardi, Raimondo Ferrara, Antonio Trogu, Rossana Berardi, Silvana Leo, Alessandro Bertolini, Francesco Angelini, Saverio Cinieri, Antonio Russo, Salvatore Pisconti, Anna Elisabetta Brunetti, Amalia Azzariti, Daniele Santini

**Affiliations:** 1 Medical Oncology Unit, National Cancer Research Centre Istituto Tumori “Giovanni Paolo II”, Bari, Italy; 2 Medical Oncology Unit, University Campus Bio-Medico, Roma, Italy; 3 Osteoncology and Rare Tumors Center, IRCCS -Romagnolo Scientific Institute for the Study and Treatment of Cancer, Meldola, Italy; 4 Medical Oncology Unit, Hospital of Frosinone, Frosinone, Italy; 5 Medical Oncology Unit, II University of Naples, Naples, Italy; 6 Medical Oncology Unit, Hospital of Latina, Latina, Italy; 7 Department of Oncology-Hematology, Hospital of Pavia, Pavia, Italy; 8 Division of Medical Oncology, Treviglio-Caravaggio Hospital, Treviglio, Italy; 9 Operative Unit of Oncology - IRCCS Fondazione Maugeri, Pavia, Italy; 10 Medical Oncology Unit, Hospital of Benevento, Benevento, Italy; 11 Department of Medical Oncology, San Giovanni Battista Hospital, Turin, Italy; 12 Department of Medical Oncology, Fondazione Poliambulanza of Brescia, Brescia, Italy; 13 Medical Oncology Unit, Hospital of Pesaro, Pesaro, Italy; 14 Medical Oncology Unit, Hospital Careggi, Florence, Italy; 15 Department of Oncology, Gradenigo Hospital and Gruppo Piemontese Sarcomi, Turin, Italy; 16 Medical Oncology Unit, Hospital “Casa Sollievo della Sofferenza”, San Giovanni Rotondo, Italy; 17 Medical Oncology Unit, Hospital “Cardarelli”, Naples, Italy; 18 Medical Oncology Unit, Hospital of Barletta, Barletta, Italy; 19 Medical Oncology Unit, Hospital of Aosta, Aosta, Italy; 20 Medical Oncology Unit, University of Ancona, Ancona, Italy; 21 Medical Oncology Unit, Hospital of Lecce, Lecce, Italy; 22 Medical Oncology Unit, Hospital of Sondrio, Sondrio, Italy; 23 Medical Oncology Unit, Hospital Regina Apostolorum, Albano Laziale, Italy; 24 Medical Oncology Department & Breast Unit – Hospital of Brindisi and Medical Oncology Department – European Institute of Oncology, Milan, Italy; 25 Medical Oncology Unit, University of Palermo, Palermo, Italy; 26 Medical Oncology Departement Hospital of Taranto, Taranto, Italy; 27 Clinical and Preclinical Pharmacology Laboratory, National Cancer Research Centre Istituto Tumori “Giovanni Paolo II”, Bari, Italy; Shanghai Jiao Tong University School of Medicine, China

## Abstract

**Background:**

Bone metastasis represents an increasing clinical problem in advanced gastric cancer (GC) as disease-related survival improves. In literature, few data on the natural history of bone disease in GC are available.

**Patients and Methods:**

Data on clinicopathology, skeletal outcomes, skeletal-related events (SREs), and bone-directed therapies for 208 deceased GC patients with evidence of bone metastasis were statistically analyzed.

**Results:**

Median time to bone metastasis was 8 months (CI 95%, 6.125–9.875 months) considering all included patients. Median number of SREs/patient was one. Less than half of the patients (31%) experienced at least one and only 4 and 2% experienced at least two and three events, respectively. Median times to first and second SRE were 2 and 4 months, respectively. Median survival was 6 months after bone metastasis diagnosis and 3 months after first SRE. Median survival in patients who did not experience SREs was 5 months. Among patients who received zoledronic acid before the first SRE, the median time to appearance of first SRE was significantly prolonged compared to control (7 months vs 4 months for control; *P*: 0.0005).

**Conclusions:**

To our knowledge, this retrospective analysis is the largest multicenter study to demonstrate that bone metastases from GC are not so rare, are commonly aggressive and result in relatively early onset of SREs in the majority of patients. Indeed, our large study, which included 90 patients treated with ZOL, showed, for the first time in literature, a significant extension of time to first SRE and increase in the median survival time after diagnosis of bone metastasis. Taken together, these data may support the beneficial effects of ZOL in GC patients.

## Introduction

Gastric cancer (GC) is the fourth most common cancer diagnosis worldwide in men following lung, prostate and colorectal, and the fifth in women following breast, colorectal, cervical and lung with an expected incidence of 640,000 and 350,000 cases in 2011, respectively [1]. Approximately 8% of total cases and 10% of annual cancer deaths worldwide are attributed to GC [2]. Curative treatment of locally confined GC is gastric resection with regional lymphadenectomy intended to remove macroscopic and microscopic disease. Conversely, when distant sites are involved, no optimal therapeutic strategy has yet been established. Almost one third of GC patients presents metastatic disease and, after curative resection, over one third of all patients will eventually develop liver-specific recurrences [3]. In addition to liver spreading, other major sites of GC metastasis are peritoneum, lungs and bone. To date, only a few studies have been conducted on the onset of bone metastases in GC, with one report focused on the topic [4]. Moreover, few international guidelines recommend to routinely evaluate bone metastasis at the time of diagnosis or during follow up or pharmacological treatment. Bone metastases in GC are mainly osteolytic impairing bone integrity and inducing bone pain. Indeed, they result in significant morbidity for patients from the associated skeletal-related events (SREs), defined as pathologic fractures, the need for radiotherapy for bone pain, surgical interventions to treat or prevent an impending fracture, spinal cord and nerve root compressions, and hypercalcemia [4]. SREs cause significant decrease of functional independence, loss of autonomy and impairment of patients' quality of life [5]. Radiotherapy seems to be the most common SRE in GC patients i.e., approximately 95% of patients receive radiotherapy, 8% of them develop pathologic fractures and another 8% require surgical decompression [4]. Despite bone metastasis causes high rates of SREs, this topic in GC has received only little attention. Early detection and availability of new primary therapies have extended patient survival, thereby leaving patients with bone metastasis at risk of SREs for a longer time.

Finally this is, to our knowledge, the largest multicenter study investigating the natural history of patients with bone metastases from GC existing in literature.

## Patients and Methods

### Ethics statement

This multicenter retrospective observational study has been approved by the Ethics Committee of the coordinator center (National Cancer Institute of Bari). According to our Ethics Committee, a written consent was not needed. In fact, this is a retrospective observational study considering only died patients whose recruitment in the survey did not influenced their treatment.

### Study design

A retrospective, observational multicenter study aimed to define the natural history of GC patients with bone metastasis was conducted in 22 Italian hospital centres in which these patients received diagnosis and treatment of disease from 1998 to 2011. Data were collected from GC patients of all ages who received standard treatments in accordance with each own treating physician's practice and were not included neither in clinical trials nor experimental protocols. Moreover, patients had at least one bone metastasis during the course of their disease and died of GC or gastric cancer-related complications. In details, patients were identified as having bone metastasis if two of the following criteria were satisfied: physician reported bone metastasis; bone metastasis identified by bone scan; record of radiotherapy to bone as a palliative therapy; identification of bone metastasis by other imaging assessment (e.g. standard x-rays, computed tomography scans, or magnetic resonance imaging of the skeleton).

Data were collected throughout the disease course and during all cancer treatments, including surgery, radiation therapy, chemotherapy, and biological therapies. Variables assessed included age, sex, histotype, number and sites of bone metastasis, nodal stage, nodal dissection, visceral metastases, ECOG performance status at the moment of bone metastases diagnosis, time to appearance of bone metastasis, times to first and subsequent SREs (from diagnosis of bone metastasis), SRE types, survival after first SRE, and type and times of bisphosphonate therapy.

### Statistical analysis

Descriptive statistics were used for patient demographics and incidence of SREs. All survival intervals were determined by the Kaplan-Meier method [6]. The differences in survival according to clinical parameters or treatment were evaluated by the log-rank test and described by the Kaplan–Meier method [7] unless otherwise specified. In the univariate model, all the clinical variables were evaluated as predictors for shorter time to bone metastasis, shorter time from bone metastases to SRE and shorter time from bone metastases to death. Patients who did not have a recorded date for a specific event were censored at the date of death. Finally, the Cox proportional hazards model was applied to the multivariate survival analysis. All the significant variables in the univariate model were used to build the multivariate model of survival, and median values were derived from whole-month values rather than fractions. SPSS software (version 20.00; SPSS, Chicago, IL) was used for statistical analysis. A *P* value <0.05 was considered statistically significant.

## Results

### Patient characteristics

The analysis of records of more than 2000 patients, died from GC, allowed to identify 208 patients (10%) with bone metastasis. 59 of them (28%) had bone metastasis at the GC diagnosis and 149 (62%) developed bone metastasis after GC diagnosis. 137/208 patients included in this study (66%) were male, consistent with the well-known male predominance of GC. The median age was 61 years. Tumor histology was intestinal in 38.9% of patients, diffuse in 33.7% and mixed plus undifferentiated in the remaining 27.4%. 81.4% of patients have been submitted to D2 node's dissection, the remaining 18.6% to D1 dissection. 86.3% of patients developed also visceral metastases (Table 1).

**Table 1:** Patient demographics and disease characteristics in the entire population.

BASELINE CHARACTERISTICS	FREQUENCY	PERCENTAGE (%)
**AGE**		
<61 YEARS (MEDIAN VALUE)	110/208	52,9
>61 YEARS (MEDIAN VALUE)	98/208	47,1
**SEX**		
MALE	138/208	66,3
FEMALE	70/208	33,7
**NODAL STATUS a**		
N0	4/142	2,8
N1	44/142	31,0
N2	59/142	41,5
N3	35/142	24,6
**HYSTOLOGY**		
INTESTINAL	74/190	38,9
DIFFUSE	64/190	33,7
OTHER	52/190	27,4
**GRADING**		
G2	29/155	18,7
G3	126/155	81,3
**NODE DISSECTION D1 VS D2**		
D1	18/97	18,6
D2	79/97	81,4
**VISCERAL METASTASIS STATUS**		
NO	28/204	13,7
YES	176/204	86,3
**ECOG PERFORMANCE STATUS b**		
0	53/187	28,3
1	82/187	43,9
2	41/187	21,9
3	11/187	5,3

a At time of diagnosis; ^b^At time of bone metastasis diagnosis.

### Skeletal metastases

**Figure 1:**
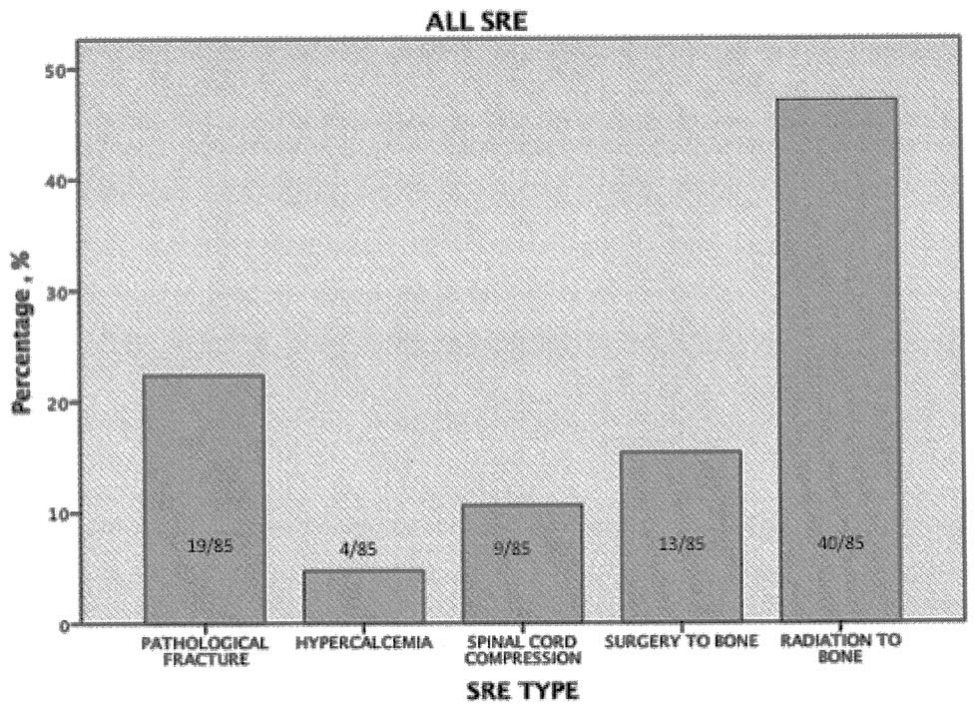
Percentage of skeletal-related events (SREs) occurring in patients with bone metastases from gastric cancer.

The majority of patients (68.6%) had multiple bone metastases and the remaining 31.4% showed single lesion. Long bones were the most common site of bone metastasis (52% of patients) followed by hip (38%) and spine (only 20% s). Osteolytic lesions (52%) were far more prevalent in this group than the mixed ones (25%) while osteoblastic lesions were not so rare as expected (23%) (Table 2). Less than half of the patients (31%) experienced at least one SRE while, two and three SREs have been reported in only 4% and 2% of patients, respectively. In Figure 1, the incidences of different SREs are reported and are consistent with previous reports i.e., radiotherapy to bone is the most common SRE (47.1% of all events), followed by pathologic fracture (22.4%), surgery to bone (15.3%) and by spinal cord compression, which accounted for 10.6% of the total number of SREs experienced in this analysis. Only 4.7% of all events is represented by hypercalcemia.

**Table 2:** Patient bone disease characteristics in the entire population.

BASELINE CHARACTERISTICS	FREQUENCY	PERCENTAGE (%)
**BONE LESION TYPE**		
OSTEOLYTIC	105/202	52,0
OSTEBLASTIC	46/202	22,8
MIXED	51/202	25,2
**BONE METASTASIS NUMBER**		
1	65/207	31,4
>1	142//207	68,6
**BONE METASTASIS SPINE**		
YES	42/207	20,3
NO	165/207	79,7
**BONE METASTASIS LONG BONES**		
YES	109/207	52,7
NO	98/207	47,3
**BONE METASTASIS HIP**		
YES	79/207	38,3
NO	127/207	61,7
**ZOLEDRONIC ACID TREATMENT**		
NO	105/186	56,5
YES	81/186	43,5
**SRE TYPE (ALL) a**		
PATHOLOGICAL FRACTURE	19/85	22,4
HYPERCALCEMIA	4/85	4,7
SPINAL CORD COMPRESSION	9/85	10,6
SURGERY TO BONE	13/85	15,3
RADIATION TO BONE	40/85	47,1

a Included first, second and third; SRE, skeletal-related event.

### Predictive factors of survival after bone metastases diagnosis

The univariate analysis, reported in Table 3, demonstrates that survival after diagnosis of bone metastases was significantly shorter in younger population (<61 years old) (p: 0.025), in patients submitted to D2 lymph nodes dissection (p: 0.009), in ECOG 2/3 patients (p: 0.002), and in patients not treated with bisphosphonates (p: 0.001). Intriguingly, in multivariate analysis (Table 4.) only D2 lymph nodes dissection independently correlates with a shorter survival after bone disease occurrence (p: 0.008; HR: 2.285).

**Table 3:** Median survival after bone metastases diagnosis: univariate analysis.

BASELINE CHARACTERISTICS		TIME (MONTHS)	95% C.I		P
			LOWER LIMIT	UPPER LIMIT	
AGE	<61 YEARS (MEDIAN VALUE)	5,000	4,165	5,835	0,025
	>61 YEARS (MEDIAN VALUE)	7,000	5,035	8,965	
SEX	MALE	6,000	4,821	7,179	0,199
	FEMALE	6,000	3,673	8,327	
HYSTOLOGY	INTESTINAL	7,000	5,656	8,344	0,136
	DIFFUSE	5,000	4,139	5,861	
	OTHER	5,000	3,766	6,234	
GRADING	G2	7,000	5,053	8,947	0,548
	G3	5,000	3,519	6,481	
HER2 STATUS	POSITIVE	5,000	4,546	5,454	0,379
	NEGATIVE	5,000	2,400	7,600	
	UNKNOW	6,000	4,856	7,144	
NODE DISSECTION D1 VS D2	D1	7,000	4,921	9,079	0,009
	D2	5,000	3,809	6,191	
VISCERAL METASTASIS STATUS	NO	5,000	1,706	8,294	0,719
	YES	6,000	5,075	6,925	
ECOG PERFORMANCE STATUS	0	7,000	5,121	8,879	0,002
	1	6,000	4,870	7,130	
	2	4,000	2,497	5,503	
	3	1,000	NA	NA	
BONE LESION TYPE	OSTEOLYTIC	6,000	4,859	7,141	0,672
	OSTEBLASTIC	6,000	3,110	8,890	
	MIXED	6,000	4,123	7,877	
BONE METASTASIS NUMBER	1	16,000	8,466	23,534	0,077
	>1	13,000	9,686	16,314	
ZOLEDRONIC ACID TREATMENT	NO	5,000	4,147	5,853	0,001
	YES	8,000	6,210	9,790	
FIRST SRE TYPE	NO SRE	5,000	3,785	6,215	0,568
	PATHOLOGICAL FRACTURE	9,000	2,987	15,013	
	HYPERCALCEMIA	4,000	NA	NA	
	SPINAL CORD COMPRESSION	8,000	0,160	15,840	
	SURGERY TO BONE	7,000	5,829	8,171	
	RADIATION TO BONE	6,000	3,673	8,327	

CI, confidence interval; *P* determined by Log-rank test; SRE, skeletal-related event.

**Table 4:** Predictive factors of survival after bone metastases diagnosis: multivariate analysis.

BASELINE CHARACTERISTICS	P	HAZARD RATIO
AGE	0,598	0,888
NODE DISSECTION D1 VS D2	0,008	2,285
ECOG PERFORMANCE STATUS	0,608	0,940
ZOLEDRONIC ACID TREATMENT	0,651	0,903

P determined by Cox proportional hazards model.

### Predictive factors of onset of bone metastasis

In univariate analysis (Table 5), the median time to the onset of skeletal disease was significantly shorter according to T stage (p<0.001) and in patients with other than intestinal and diffuse hystology (p: 0.007), with G3 tumor (p: 0.026), and who had undergone to D2 lymph nodes dissection (p: 0.026). The multivariate analysis (Table 6) shows that only D2 lymph nodes dissection independently correlated with a shorter time to diagnosis of bone metastases (p: 0.013; HR: 2.708).

**Table 5:** Median time to bone metastases diagnosis: univariate analysis.

BASELINE CHARACTERISTICS		TIME (MONTHS)	95% C.I		P
			LOWER LIMIT	UPPER LIMIT	
AGE	<61 YEARS (MEDIAN VALUE)	6,000	2,065	9,935	0,490
	>61 YEARS (MEDIAN VALUE)	10,000	7,555	12,445	
SEX	MALE	9,000	6,500	11,500	0,386
	FEMALE	8,000	5,034	10,966	
TUMOR STAGE	T1	65,000	19,911	110,089	<0,001
	T2	22,000	18,375	25,625	
	T3	9,000	5,569	12,431	12,431
	T4	6,000	3,143	8,857	
NODAL STATUS a	N0	65,000	,630	129,370	0,142
	N1	10,000	6,843	13,157	
	N2	11,000	7,743	14,257	
	N3	12,000	2,758	21,242	
HYSTOLOGY	INTESTINAL	9,000	6,330	11,670	0,007
	DIFFUSE	10,000	7,425	12,575	
	OTHER	5,000	1,491	8,509	
GRADING	G2	10,000	0,000	25,904	0,026
	G3	9,000	6,689	11,311	
NODE DISSECTION D1 VS D2	D1	21,000	12,684	29,316	0,026
	D2	16,000	10,468	21,532	
VISCERAL METASTASIS STATUS	NO	5,000	0,000	15,371	0,279
	YES	8,000	6,203	9,797	
BONE LESION TYPE	OSTEOLYTIC	8,000	5,681	10,319	0,257
	OSTEBLASTIC	15,000	5,321	24,679	
	MIXED	7,000	4,440	9,560	

a At time of diagnosis; CI, confidence interval; *P* determined by Log-rank test; SRE, skeletal-related event.

**Table 6:** Predictive factors of time of bone metastases diagnosis: multivariate analysis.

BASELINE CHARACTERISTICS	P	HAZARD RATIO
AGE	0,927	1,023
TUMOR STAGE	0,145	1,351
HISTOLOGY	0,549	1,110
GRADING	0,519	0,794
NODE METASTASIS STATUS	0,193	1,389
NODE DISSECTION D1 VS D2	0,013	2,708

*P* determined by Cox proportional hazards model.

### Skeletal outcomes and SREs in the overall population

All GC patients included in this study (*N* = 208) showed a median overall survival time of 14 months (CI 95%, 12.025–15.975) and a median time to diagnosis of bone metastasis of 8 months (CI 95%, 6.125–9.875 months). The median level of maximum bone pain experienced after diagnosis of bone metastasis was 8 months (range, 0–10) while, the median pain level experienced at the time of diagnosis was 5 months (range, 0–9). At the time of bone metastases diagnosis, 27% of patients showed an ECOG Performance Status of 2 or 3. The median number of SREs experienced by patients was one (range, 0–3). The median time to first SRE after confirmed diagnosis of bone metastasis was 2 months (CI 95%, 1.536–0–2.464 months), indicative of the severity of bone metastasis in GC. The median time to second SRE was 4 months (CI 95%,3.457–4.865 months). Median survival from the diagnosis of bone metastasis was 6 months (CI 95%,5.068–6.932 months). Median survival after development of the first SRE was 3 months (CI 95%,2.049–3.951 months). Median survival in patients who did not experience SREs was 5 months (CI 95%, 3.785–6.125 months). All data described are reported in Table 7.

**Table 7:** Patients survival parameters according bone metastasis onset.

PATIENTS SURVIVAL PARAMETERS	TIME, MO	95% C.I
**ALL PATIENTS**		
OVERALL SURVIVAL	14	12,025–15,975
SURVIVAL AFTER BONE METASTASIS DIAGNOSIS	6	5,068–6,932
TIME TO SRE AFTER BONE METASTASIS DIAGNOSIS	2	1,536–2,464
SURVIVAL AFTER SRE	3	2,049–3,951
TIME TO BONE METASTASIS	8	6,125–9,875
**BONE METASTASIS SYNCRHONOUS**		
SURVIVAL AFTER BONE METASTASIS DIAGNOSIS	5	3,461–6,539
TIME TO SRE AFTER BONE METASTASIS DIAGNOSIS	1	0,530–1,470
SURVIVAL AFTER SRE	4	3,156–4,844
**BONE METASTASIS METACHRONOUS**		
SURVIVAL AFTER BONE METASTASIS DIAGNOSIS	5	3,830–6,170
TIME TO SRE AFTER BONE METASTASIS DIAGNOSIS	2	1,333–2,667
SURVIVAL AFTER SRE	3	2,074–3,000
**ONLY BONE METASTASIS**		
SURVIVAL AFTER BONE METASTASIS DIAGNOSIS	5	1,679–8,321
TIME TO SRE AFTER BONE METASTASIS DIAGNOSIS	1	0,000–2,283
SURVIVAL AFTER SRE	3	1,829–4,171

CI, confidence interval; SRE, skeletal-related event.

### Skeletal outcomes and SREs according to time of bone metastases appearance

The entire population was divided in three subpopulations (synchronous bone metastases, metachronous bone metastases and patients with only bone metastases) and each subgroups was characterised for the following parameters: clinical, pathological and bone metastases characteristics, SREs and skeletal outcomes. Any significant difference was found in terms of age, gender, histology, visceral metastases, type, site and number of bone lesions. Intriguingly, the majority of patients (66,7%) with only bone metastases experienced, as the more frequent SRE, radiation to bone compared with 55,6% and 52,2% of patients with synchronous and metachronous metastases, respectively. Interestingly, median survival after bone metastases diagnosis resulted the same (5 months) in the three groups of patients, indicative of the poor prognosis strictly related to the presence of bone disease in GC patients. All data are summarised in Table 8.

**Table 8:** Skeletal outcomes and SRE according to time of bone metastases appearance.

BASELINE CHARACTHERISTICS	BONE METASTASIS SYNCRHONOUS		BONE METASTASIS METACHRONOUS		ONLY BONE METASTASIS	
	FREQUENCY	(%)	FREQUENCY	(%)	FREQUENCY	(%)
**AGE**						
<61 YEARS (MEDIAN VALUE)	35\59	59,3	75\149	50,3	16\28	57,1
>61 YEARS (MEDIAN VALUE)	24\59	40,7	74\149	49,7	12\28	42,9
**SEX**						
MALE	38\59	64,4	100\149	67,1	22\28	78,6
FEMALE	21\59	35,6	49\149	32,9	6\28	21,4
**HYSTOLOGY**						
INTESTINAL	17\51	33,3	57\139	41,0	8\24	33,3
DIFFUSE	14\51	27,5	50\139	36,0	6\24	25,0
OTHER	20\51	39,2	32\139	23,0	10\24	41,7
**VISCERAL METASTASIS STATUS**						
NO	9\59	15,3	19\145	13,1	28\28	100,0
YES	50\59	84,7	126\145	86,9	0\28	0,0
**BONE LESION TYPE**						
OSTEOLYTIC	32\58	55,2	73\144	50,7	13\27	48,1
OSTEBLASTIC	7\58	12,1	39\144	27,1	7\27	25,9
MIXED	19\58	32,8	32\144	22,2	7\27	25,9
**BONE METASTASIS NUMBER**						
1	15\59	25,4	50\148	33,8	8\28	28,6
>1	44\59	74,6	98\148	66,2	20\28	71,4
**BONE METASTASIS SPINE**						
YES	8\58	13,8	34\149	22,8	4\28	14,3
NO	50\58	86,2	115\149	77,2	24\28	85,7
**BONE METASTASIS LONG BONES**						
YES	27\58	46,6	82\149	55,0	10\28	35,7
NO	31\58	53,4	67\149	45,0	18\28	64,3
**BONE METASTASIS HIP**						
YES	19\57	33,3	60\149	40,3	11\28	39,3
NO	38\57	66,7	89\149	59,7	17\28	60,7
**BISPHOSPHONATE**						
ZOLEDRONIC ACID	27\59	45,8	58\149	38,9	13\28	46,4
OTHER	3\59	5,1	7\149	4.7	0\28	0,0
NO BISPHOSPHONATE	5\27	49,2	84\159	56,4	15\28	53,6
**FIRST SRE TYPE**						
PATHOLOGICAL FRACTURE	2\27	18,5	9\46	19,6	1\19	11,1
HYPERCALCEMIA	2\27	7,4	1\46	2,2	1\19	11,1
SPINAL CORD COMPRESSION	3\27	7,4	4\46	8,7	1\19	11,1
SURGERY TO BONE	5\27	11,1	8\46	17,4	0\19	0,0
RADIATION TO BONE	15\27	55,6	24\46	52,2	6\19	66,7

SRE, skeletal-related event.

### Bisphosphonate therapy

**Figure 2:**
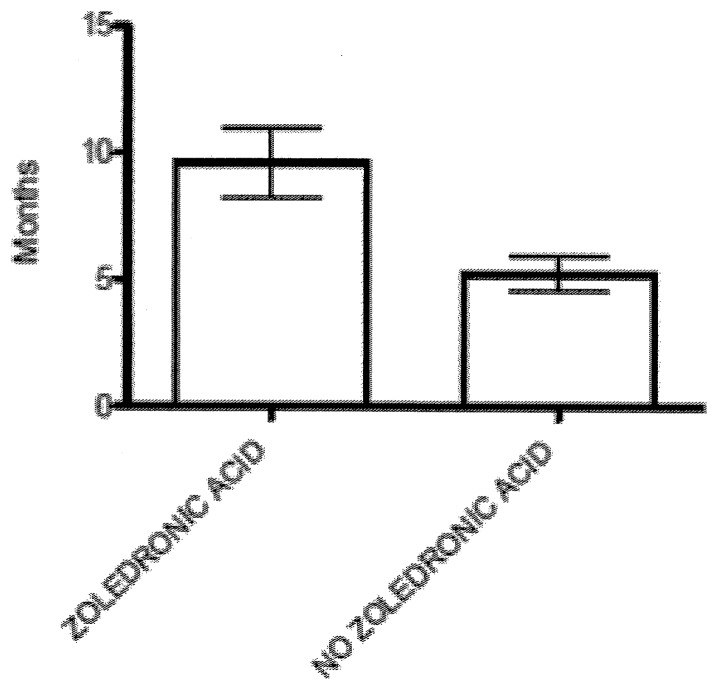
Comparison of time to first SRE in patients receiving zoledronic acid before SRE (n = 31) and those who did not receive zoledronic acid (n = 85, p = 0.0005). Data are presented as mean ± SEM Statistical significance was determined by Mann-Whitney test.

Among the 208 patients with bone metastasis, 43.5% were treated with zoledronic acid (ZOL) (administrated at a dose of 4 mg every 4 weeks via 15-minute infusion, with dose adjustments based on creatinine clearance), 3.4% received pamidronate (administered at a dose of 90 mg every 4 weeks via 2-hour infusion), and 53.1% did not receive any bisphosphonate treatment (Table 2). ZOL was generally well tolerated; only one patient developed osteonecrosis of the jaw (ONJ). Patient with ONJ underwent a computed tomography scan for confirmation; no retrospective adjudication was performed. It should be noted that no preventive dental care was offered before bisphosphonate therapy because many patients included in the study received treatment before 2006. Patients receiving ZOL treatment had a longer median survival time after diagnosis of bone metastasis compared with patients naives for treatment with bisphosphonates (8 months [CI%, 6.210–9.790 months] versus 5 months [CI%, 4.147–5.853 months], respectively) (*P*: 0.001, Table 3). In addition, patients who received ZOL before the onset of SRE (31 patients) have experienced it, after the diagnosis of bone metastases, in a time statistically higher than patients who did not receive treatment with bisphosphonates (7 months [CI%, 6.790–12.430 months] versus 4 month [CI%, 3.870–6.600 months], *P*: 0.0005). (Figure 2).

## Discussion

To our knowledge, this study is the largest multicenter survey investigating the natural history of metastatic bone disease in patients with GC. Bone metastasis was confirmed in approximately 10% of our screened GC patients, and this percentage is quite higher than expected [8]–[11]. In the present study, all patients with at least one known bone metastasis were included, conversely, several patients with poorly documented bone metastasis were omitted. Moreover, patients with documented bone metastases, but who were alive at study entry, were excluded. The high number of included patients and the restrictive inclusion criteria support the reliability of this incidence. Among the 10% of GC patients with bone metastasis, approximately one third presented it at the time of initial GC diagnosis, whereas the others developed bone metastasis during disease progression. Interestingly, median survival after bone metastases diagnosis resulted the same in both the study groups (5 months). Moreover, these two populations of bone metastatic GC patients did not shown any significant difference in terms of clinical, pathological and bone metastases characteristics, SREs and skeletal outcomes. The lack of outcome differences could be indicative of the poor prognosis linked to bone disease in GC patients. Only half of bone lesions were lytic and the blastic lesions (23%) were not so rare as previously reported in literature [12], [13]. The axial skeleton was involved in only 20% of GC patients included in the survey, the frequency is lower than that observed in breast cancer (83%) [14] or reported by Park in GC (86%) [15]. Patients showed the majority of metastatic sites in the long bones (52%), followed by hip (38%) and spine. However, sites of metastatic growth may be governed by the mechanism of metastasis.

Among all the clinical and pathological parameters correlated with median time to diagnosis of bone metastasis and median survival after skeletal disease appearance, only D2 lymph nodes dissection, at multivariate analysis, resulted independently correlated with both outcomes. Moreover, in univariate analysis, nodes staging did not correlate neither with time to skeletal disease, nor with survival after bone disease (data not shown). There is not a clear clinical or biological rational for explaining these correlations. It's clearly demonstrated in literature that in GC the number of examined nodes is a strong independent predictors of better survival [16]–[18] and, no data have been reported on a potential detrimental effect of an extensive lymphadenectomy. With the actual knowledge, we may only hypothesize on a potential cancer cell “bone spreading effect” triggered by D2 dissection. The median survival time of 6 months after diagnosis of skeletal metastasis in our study population is higher than that previously reported in literature [12]. For this reason the majority of these patients may experience extremely debilitating skeletal complications (i.e. SREs) that profoundly impact their quality of life. Median survival after SRE occurrence was only 3 months, possibly because of aggressive SREs affecting survival, or other complications related to SREs. The median time to diagnosis of bone metastasis was 8 months and the median time to first SRE was very short (only 2 months), thereby highlighting the need for effective bone-targeted therapy aiming to delay bone metastasis appearance and SREs. Bisphosphonates (such as ZOL, pamidronate, and clodronate) are highly effective inhibitors of osteoclast-mediated bone resorption and have been widely used for treating and preventing SREs from bone metastases in solid tumors and multiple myeloma [18]–[22]. ZOL is the only bisphosphonate with approved efficacy in all solid tumors. Perspective data on the efficacy of bisphosphonates in bone metastatic gastric cancer are lacking in literature. More recently, the receptor activator of nuclear factor kappa-B ligand inhibitor denosumab has also shown broad efficacy for SRE reduction in patients with bone metastasis from solid tumors [23] however, denosumab was not available outside of a clinical trial during the period spanned by our retrospective database analysis. Our large study, which included 90 patients treated with ZOL, showed, for the first time in literature, a significant extension of time to first SRE and increase in the median survival time after diagnosis of bone metastasis. Taken together, these data may support the beneficial effects of ZOL in GC patients. This findings are in accordance with recently published data showing that zoledronic acid treatment significantly prolong median time to first SRE and lead as well to a trend toward an improved overall survival in bone metastatic colorectal cancer patients [24]. Additionally, although intravenous bisphosphonates have been associated with dose- and infusion rate-dependent decreases in renal function [25], in the current study the renal safety profile of ZOL in GC was similar to the renal safety profile in patients not treated with bisphosphonates.

Limitations of this study include its retrospective design and inclusion of an unselected heterogeneous cohort of patients with all types of histologic variants of GC, as well as a range of anticancer therapies. However, the types of patients included in this study represent the typical scenario of a real clinical practice. Another limitation of a chart review is the heterogeneity of standardized methods used for detecting bone metastases, with each methodology having its own limit of detection.

To our knowledge, this retrospective analysis is the largest multicenter study to demonstrate that bone metastases from GC are not so rare, are commonly aggressive and result in relatively early onset of SREs in the majority of patients.
